# Vitamin D in Osteosarcopenic Obesity

**DOI:** 10.3390/nu14091816

**Published:** 2022-04-26

**Authors:** Luigi Di Filippo, Rebecca De Lorenzo, Andrea Giustina, Patrizia Rovere-Querini, Caterina Conte

**Affiliations:** 1 School of Medicine, Vita-Salute San Raffaele University, Via Olgettina 58, 00132 Milan, Italy; difilippo.luigi@hsr.it (L.D.F.); delorenzo.rebecca@hsr.it (R.D.L.); giustina.andrea@hsr.it (A.G.); rovere.patrizia@hsr.it (P.R.-Q.); 2 Institute of Endocrine and Metabolic Sciences, IRCCS San Raffaele Hospital, Via Olgettina 60, 00132 Milan, Italy; 3 Division of Immunology, Transplantation and Infectious Diseases, IRCCS San Raffaele Hospital, Via Olgettina 60, 00132 Milan, Italy; 4 Department of Human Sciences and Promotion of the Quality of Life, San Raffaele Roma Open University, Via di Val Cannuta 247, 00166 Rome, Italy; 5 Department of Endocrinology, Nutrition and Metabolic Diseases, IRCCS MultiMedica, Via Milanese 300, Sesto San Giovanni, 20900 Milan, Italy

**Keywords:** obesity, vitamin D, insulin resistance, sarcopenic obesity, sarcopenia, osteopenia, osteoporosis

## Abstract

Osteosarcopenic obesity is a unique clinical condition where low bone and muscle mass coexist in individuals with obesity. Alterations in adipose tissue, skeletal muscle and bone are strictly interconnected, and vitamin D plays key roles in several metabolic pathways that are involved in maintaining musculoskeletal health and glucose homeostasis. We reviewed the available literature on mechanisms underlying osteosarcopenic obesity, with a focus on the role of vitamin D in the pathogenesis and treatment of the condition. We found that, although evidence from large observational studies and pre-clinical experiments strongly supports a role of vitamin D deficiency in the pathogenesis of osteosarcopenic obesity, the common belief that vitamin D improves musculoskeletal health lacks solid clinical evidence, as trials specifically aimed at assessing the effects of vitamin D supplementation in patients with osteosarcopenic obesity are not available, and trials that investigated the role of vitamin D on muscle and bone health in other patient populations either showed no or even detrimental effects. We conclude that large observational and interventional studies including individuals with osteosarcopenic obesity representative of different sex, age and race are needed to better define the role of vitamin D in the pathogenesis and treatment of this condition.

## 1. Introduction

Sarcopenic obesity is now recognized as a specific clinical entity characterized by excess fat mass (obesity) with reduced skeletal muscle mass and function (sarcopenia) [1]. Several metabolic alterations occur in the skeletal muscle of individuals with obesity that may negatively impact muscle mass and function. Inflammation and oxidative stress exert catabolic effects and may induce anabolic resistance in skeletal muscle, ectopic fat accumulation with fatty infiltration (myosteatosis) results in lipotoxicity, alterations in muscle stem cell may determine a shift towards adipocyte differentiation, and mitochondrial dysfunction leads to less efficient energy production and oxidative stress [2]. With the progression of obesity, functional limitations imposed by increasing body mass and musculoskeletal complications may further worsen skeletal muscle and bone health. The latter is also affected by the metabolic alterations and the proinflammatory *milieu* associated with obesity. For a long time, obesity has been thought to have favorable effects on bone, due to the anabolic stimulus of mechanical load [3] and estrogen levels [4] associated with excess fat mass. However, recent evidence suggests that, despite increased bone mineral density (BMD), fracture risk is increased at specific skeletal sites in individuals with obesity [5]. In older adults with sarcopenic obesity, femoral neck BMD was lower than in older adults with obesity alone [6]. These observations confirm previous evidence of reduced BMD in subjects with sarcopenic obesity as compared to obesity alone, and support the concept that sarcopenia may increase the risk of low bone mass and fracture risk [7,8], as an increase in muscle mass parallel to the increase in fat mass appears to be necessary for BMD to increase [9]. It appears clear that alterations in adipose tissue, skeletal muscle and bone are strictly interconnected. The term “osteosarcopenic obesity” was introduced to describe the co-existence of low bone and muscle mass in individuals with obesity [10,11], although an univocal consensus on diagnostic criteria is still lacking [12], as was for sarcopenic obesity until recently [1]. Osteosarcopenic obesity is often underrecognized, despite being associated with poor functional and metabolic outcomes [13]. Vitamin D is a micronutrient that is essential to musculoskeletal health [14] but also exerts extra-musculoskeletal functions, being involved in several metabolic pathways [15]. Its deficiency possibly plays a role in the pathogenesis of osteosarcopenic obesity, but whether affected patients would benefit from vitamin D supplementation is uncertain. The aim of this narrative review is to provide an overview of the mechanisms underlying osteosarcopenic obesity, with a focus on the role of vitamin D in the pathogenesis and treatment of the condition.

## 2. Osteosarcopenic Obesity: Prevalence, Risk Factors and Impact on Health Outcomes

Prevalence estimates of osteosarcopenic obesity vary widely depending on the population and the definition used, ranging from ~0% to 19% [16,17,18,19] and increasing with age [19], although reduced bone and skeletal muscle mass have been reported even in young (18–21 years old) subjects with overweight/obesity [20]. The risk of osteosarcopenia increases with increasing fat mass: prevalence ratios of 1.46 (95% CI 1.11 to 1.92) and 2.25 (95% CI 1.71 to 2.95), respectively, were found in men and women aged 60 years or older [21]. In keeping with these findings, subjects with the metabolic syndrome, which is almost invariably associated with excess adiposity and insulin resistance, were found to have a ~2.5-fold risk of having osteosarcopenic obesity as compared with controls [22]. Age, sex, race and excess alcohol consumption are all factors associated with increased odds of having osteosarcopenic obesity [19]. Dietary factors have also been implicated, such as low calcium [23] and protein [24] intake, poor diet quality [25], and higher dietary inflammatory index [26].

It is possible that specific populations are at increased risk of osteosarcopenic obesity due to conditions or treatments associated with loss of skeletal muscle and bone alongside gains in adipose tissue. As examples, patients with Cushing’s disease due to excess glucocorticoid exposure [27], patients with type 2 diabetes, as type 2 diabetes is associated both with overweight/obesity and poor bone quality [28,29] and insulin resistance/deficiency may contribute to sarcopenia [30], kidney transplant recipients, who often gain weight and are treated with long-term steroid therapy after transplantation [31], as well as patients with chronic obstructive pulmonary disease, human immunodeficiency virus, cancer [11], and—possibly—patients who survived severe forms of COVID-19, due to high-dose steroid treatment and increased adiposity due to disproportional abdominal fat regain following weight loss in the acute phase [32]. Although no specific studies have been conducted so far in these populations, it is likely that the prevalence of osteosarcopenic obesity is high.

Osteosarcopenic obesity strongly impacts several health outcomes. Skeletal muscle is the major site of insulin-stimulated glucose utilization. It is not surprising that reduced muscle mass in obesity is strongly associated with insulin resistance and altered glucose metabolism, particularly in the elderly [33,34]. Although the association between sarcopenic obesity and insulin resistance is widely recognized [35], few studies so far have assessed the relationship between osteosarcopenic obesity and insulin resistance. In non-diabetic Korean men and postmenopausal women aged 50 years or older, the odds for insulin resistance were highest in subjects with obesity or osteopenic obesity, followed by sarcopenic obesity and osteosarcopenic obesity, whereas those without obesity had the lowest risk [36]. Another Korean study in subjects with obesity aged 50 years or older from the Korea National Health and Nutrition Examination Survey found that women with osteosarcopenic obesity had significantly greater insulin resistance (HOMA-IR 3.3) than those with obesity (HOMA-IR 2.6) or osteopenic obesity (HOMA-IR 2.8), and similar to those with sarcopenic obesity (HOMA-IR 2.9), whereas, among men, those with sarcopenic obesity had the greatest insulin resistance [37]. Associations have also been reported between osteosarcopenic obesity, the metabolic syndrome [37] and its individual components, namely dyslipidaemia [38] and arterial hypertension [37,39]. Both insulin resistance and the metabolic syndrome have been associated with frailty risk [40,41], and may therefore further aggravate the burden of osteosarcopenic obesity on functional status. A study in Chinese community-dwelling elderly individuals found that those with osteosarcopenic obesity had significantly lower physical performance, as assessed by the timed up-and-go test, as compared with obesity alone [42]. Postmenopausal women with osteosarcopenic obesity have significantly lower handgrip strength, slower normal and brisk walking speed, and shorter time for each leg stance than women with obesity alone [17]. Within subjects with osteosarcopenic obesity, those with predominantly increased visceral fat appear to be at higher risk of fracture as compared with those in whom subcutaneous fat predominates [43], highlighting once again the key role of visceral adiposity and related inflammation. Overall, osteosarcopenic obesity is associated with poor functionality, and might thus increase the risk of frailty.

## 3. Pathogenic Mechanisms Underlying Osteosarcopenic Obesity

### 3.1. Role of Endocrine Dysregulation

**Figure 1:**
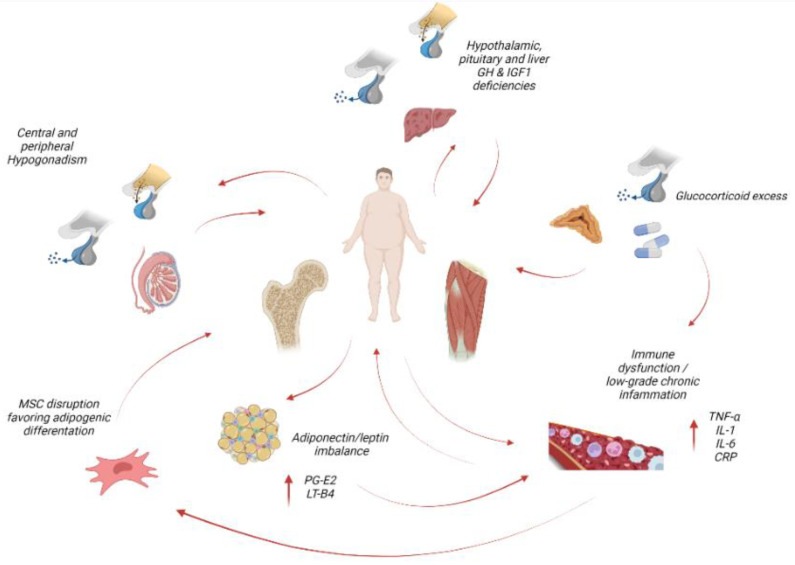
Role of endocrine and inflammatory/immune dysregulation in the pathogenesis of osteosarcopenic obesity. Red, curved arrows: negative influence; bidirectional red curved arrows indicate mutual influences perpetuating the pathogenic vicious cycle. Upward arrows indicate an increase. Causes of glucocorticoid excess include Cushing disease/syndrome, aging, sleep deprivation, stress, steroid therapy; ageing is associated with GH, IGF1 and androgen deficiency; adipose tissue dysfunction triggers low-grade chronic inflammation. See text for detailed explanation. GH, growth hormone; IGF1, insulin-like growth factor 1; TNF-α, tumor necrosis factor-alpha; IL-1, interleukin 1; IL-6, interleukin 6; CRP, C-reactive protein; PG-E2, prostaglandin E_2_; LT-B4, leukotriene B4. Created with BioRender.com, accessed on 31 March 2022.

The endocrine system is considered as one of the main regulating actors in obesity, sarcopenia and sarcopenic obesity [44]. Endocrine dysregulation, through complex inter-relationships with the hypothalamic–pituitary–adrenal (HPA) axis, brain, immune system, skeletal muscle and adipose tissue, may negatively influence body composition with deleterious systemic consequences (Figure 1).

*Glucocorticoids (GCs)*, in addition to the well-known effects on immune system and stress regulation, are also known to influence skeletal muscle, bone health and adipose tissue [45,46]. Elevated GC levels lead to muscle atrophy, bone damage with secondary osteoporosis, and increase of abdominal and visceral adiposity [47,48,49]. In line with these findings, several studies indicate that chronically elevated cortisol levels, possibly due to aging, sleep deprivation and stress, and an impairment of the HPA axis feedback regulation, are strictly associated with frailty in older male and female patients and poorer health outcomes in later life [50,51,52,53]. Elevated cortisol levels have been reported in elderly subjects with sarcopenic obesity [54], but also in young subjects with overweight/obesity, who exhibited greater fat mass and lower muscle and bone mass as compared with normal weight subjects [20]. These findings highlight that osteosarcopenic features may be detected even in young individuals with overweight/obesity, and suggest that increased cortisol levels may play a role in determining these alterations.

*Human growth hormone (GH) and insulin-like growth factor-1 (IGF-1)* are key determinants of body composition, exerting anabolic effects on muscle mass, decreasing total and visceral body fat mass and favoring osteoblastogenesis, bone formation and chondrogenesis. Serum GH levels decline with aging, reaching a nadir at the sixth decade, with older individuals exhibiting a daily GH secretion ranging from 1/5 to 1/20 of that observed in young adults [55]. A negative correlation between either GH or IGF-1 and the ratio between truncal fat mass and appendicular skeletal muscle mass has been reported in subjects with obesity, suggesting that altered GH/IGF-1 status may play a role in determining the sarcopenic obesity phenotype [56].

*Androgens* have several beneficial effects on skeletal muscle mass, adiposity and bone health increasing muscle protein synthesis, lipolysis and bone formation [57,58,59]. Testosterone levels decrease with advancing age, falling by around 2% of bioavailable hormone levels per year [58]. A bidirectional mechanism regulates the strict relationship between testosterone and fat mass: low testosterone levels lead to an increase in adipose tissue and, in turn, adipose tissue—especially visceral fat, converts testosterone to estradiol due to augmented aromatase enzyme expression and activity, reducing testosterone bioavailable circulating levels [60]. Several studies have shown that the correction of testosterone levels in men with obesity and in men with normal to low testosterone levels was associated with a reduction of body weight, waist circumference, visceral fat mass and a simultaneous increase of muscle mass [61,62,63,64]. Moreover, a recent placebo-controlled trial on testosterone treatment for 1 year in older men with low testosterone levels showed, compared to placebo, a significant increase of volumetric BMD and estimated bone strength [65]. Thus, a testosterone-replacement treatment could be considered for elderly patients with obesity and low levels of testosterone, as this treatment option appears to be associated with adequate cardiovascular safety [66].

### 3.2. Low-Grade Chronic Inflammation

Mechanisms underlying osteosarcopenic obesity reflect the multiple pathophysiological facets of the organism’s response to stress, which ultimately leads to metabolic derangements and dysfunctional body composition. Distinguishing the effects of acute and chronic stress on body homeostasis is paramount to fathom osteosarcopenic obesity. While acute stress prompts a flexible adaptation in a synchronous and self-resolving way [67], chronic stress induces maladaptive, yet self-perpetuating processes that culminate in persistent homeostatic imbalance [67,68]. The HPA axis is a major actor in the response to stress, its activation leading to different downstream effects depending on the type and duration of the insult [69]. Acute stress results in a rapid surge of energy through the secretion of catecholamines from the adrenal medulla and cortisol from the adrenal cortex, with beneficial hormonal, neural and immune consequences aimed for survival [70]. Chronic stress, instead, disrupts the physiological circadian fluctuations in cortisol release and causes constantly high or low serum cortisol levels [71]. The resulting prolonged, steady stimulation of glucocorticoid receptors located on immune cells leads to their progressive desensitization, with profound repercussions on immune equilibrium. Specifically, local and/or systemic inflammatory molecules, including nuclear factor kappa-beta (NF-kB), tumor necrosis factor (TNF)-alpha, and pro-inflammatory interleukins (i.e., IL-1, IL-6), become overexpressed in the absence of the regulatory control of cortisol on immune cells, ultimately leading to increased inflammation [71]. As expected, inflammation induced by chronic stress is in itself chronic, as both processes influence and perpetuate each other. Sources of chronic stress include diet and lifestyle, impaired psychological status, chronic diseases, etc. [68]. Therefore, in light of the aforementioned considerations, all these factors also lead to low-grade chronic inflammation, which in turn foments chronic stress as in a vicious cycle. Low-grade chronic inflammation is defined as the persistent increase in pro- and anti-inflammatory mediators or immune cells in the circulation [72]. A dysregulation of resolvins and protectins, key mediators of inflammation resolution, has been implicated in low-grade chronic inflammation [73]. Body composition fits into these processes as both the influencer and influenced, through several interconnected mechanisms. The inflammatory effects of excess and dysfunctional adiposity, as in patients with obesity, parallel the accumulation of more body fat through a positive feedback loop, suggesting that adipose tissue plays a major role in low-grade chronic inflammation [10,72,74]. Pro-inflammatory lipid mediators, especially prostaglandin (PG) E_2_ and leukotriene (LT) B4, induce the activation of peroxisome proliferator-activated receptor gamma (PPAR g) signaling, promoting further adipocyte proliferation [68]. The resulting increased adipose metabolism, in turn, fuels the production of inflammatory cytokines and perpetuates low-grade chronic inflammation. Accordingly, abnormal circulating levels of TNF-α, IL-6, C-reactive protein (CRP) and leptin have been found in individuals with obesity, reflecting their overexpression in adipose tissue [75,76]. Adiponectin and leptin, adipocyte-produced adipokines, have opposing effects, the first bearing anti-inflammatory properties [77], the latter being pro-inflammatory [78]. Obesity is characterized by a decreased adiponectin/leptin ratio, which has been recently suggested as an estimator of dysfunctional adipose tissue [79,80]. The actions of leptin are not limited to energy metabolism but extend to bone formation through hypothalamic interactions, with anti-osteogenic effects under certain conditions [81]. In bone, low-grade chronic inflammation stimulates osteoclastogenesis through the action of proinflammatory cytokines such as IL-6, IL-1 and TNF-α, which are produced by stressed adipocytes and adipose tissue-resident macrophages [82]. Another important mechanism of obesity-associated osteopenia involves mesenchymal stem cells (MSC), precursors of both osteoblasts and adipocytes. Low-grade chronic inflammation may disrupt MSC lineage commitment, favoring adipogenic differentiation at the expense of osteoblastogenesis [10,74]. Upregulated proinflammatory cytokines in patients with obesity may also induce osteopenia and osteoporosis by regulating the receptor activator of nuclear factor kappa-B (RANK)/RANK ligand/osteoprotegerin pathway, with a resulting decreased expression of osteoprotegerin and increased RANK signaling, culminating in osteoclastogenesis activation and bone resorption [10,83].

Obesity and obesity-related low-grade chronic inflammation deeply impact skeletal muscle health. Elevation of circulating inflammatory cytokines is associated with decreased muscle mass in the elderly [84]. Moreover, having MSC also myogenic potential, MSC lineage commitment dysfunction leading to increased adipogenesis induced by low-grade chronic inflammation is associated also to muscle tissue loss (i.e., sarcopenia) in addition to osteopenia [10]. Besides the deleterious action on MSC, obesity-associated low-grade chronic inflammation, through the increase in proinflammatory mediators and the activation of immune-mediated mechanisms, may lead to a progressive decrease in muscle mass paralleled by a shift in tissue composition towards fat deposition and accumulation in muscle due to increased insulin resistance [85]. Both reduced muscle mass and fatty infiltration of muscle (i.e., myosteatosis) may affect bone health. Low appendicular muscle mass and muscle strength have been associated with a more rapid decrease in total volumetric BMD and cortical bone parameters evaluated by HR-pQCT in older men [86,87], and myosteatosis of paraspinal muscles was inversely associated with lumbar BMD in middle-aged and elderly men and women [88]. Furthermore, the progressive physical inactivity that typically characterizes patients with obesity increases adipose tissue and decreases muscle mass even further, exacerbating metabolic dysfunction [85]. Decreased mobility also decreases bone formation, disrupting body composition thoroughly in a positive feedback loop.

## 4. Hypovitaminosis D and Osteosarcopenic Obesity

### 4.1. Vitamin D: Brief Overview

Vitamin D is a fat-soluble steroid hormone crucial for skeletal health and intestinal calcium absorption, demonstrated to prevent osteomalacia/rickets and also has many other extra-skeletal systemic actions [89]. In humans, vitamin D is prevalently produced in the skin after the exposure to sunlight as a result of irradiation of 7-dehydrocholesterol (pro- vitamin D), normally present in the skin, by UV-B rays [90,91]. UV-B irradiation of 7-dehydrocholesterol promotes a photochemical cleavage generating the pre- vitamin D3 hormone that, through a temperature-dependent molecular rearrangement, in the following 48 h is transformed into vitamin D3 (called cholecalciferol), and into tachysterol and luminosterol, two biologically inert products. Dietary intake provides not more than 20% of daily vitamin D requirements, the major dietary sources being represented by fortified cereal and dairy products, egg yolks, mushrooms, fish oils, and plants (the latter are a source of vitamin D in the vitamin D2 form, called ergocalciferol, a vitamin D form activated equally efficiently by the vitamin D–hydroxylases). Vitamin D is secreted as a pro-hormone, thereafter it is transferred into the circulation by binding to the serum vitamin D-binding protein (DBP) [92]. Approximately 90% of vitamin D circulates bound to the VDBP, 0.03% is free, and the rest 10% circulates bounding to the albumin. To obtain the biologically active form of vitamin D, a succession of two hydroxylation reactions of VD3 is required. The first hydroxylation step occurs in the liver by the VD-25-hydroxylase (CYP2R1), which converts cholecalciferol into 25-hydroxycholecalciferol (25(OH)D); the second hydroxylation step occurs in the kidney proximal tubules by the vitamin D-1-hydroxylase (CYP27B1), which converts 25-hydroxycholecalciferol into 1,25-dihydroxycholecalciferol (called calcitriol), the active form of vitamin D. This latter mechanism is under the strict control of endogenous parathyroid hormone (PTH) action, serum calcium, phosphates, and the fibroblast growth factor-23 (FGF-23), whereas the mechanisms regulating the first hydroxylation step are poorly understood [93,94,95].

The vitamin D receptor (VDR) is widely expressed on several organs and tissues, and a very high number of genes (about 3% of the human genome) are under the indirect or direct control of vitamin D in its active form, suggesting a widely ranging in the biological spectrum of vitamin D activities. In fact, vitamin D is crucial for skeletal and calcium homeostasis, and has also many extra-skeletal actions [89], including immunomodulation and body composition regulation. Assessment of total 25OH-vitamin D is widely accepted as a marker of the vitamin D status, with 25OH-vitamin D levels below 20 ng/mL representing deficiency and levels above 30 ng/mL sufficiency [95]. Vitamin D deficiency is a widespread health issue worldwide and as many as one-third of the world’s population is deficient, ranging from <20% of the population in Northern Europe, to 30–60% in Western, Southern and Eastern Europe and up to 80% in Middle East countries [96]. Several physiological and pathological conditions are known to negatively influence vitamin D production and metabolism, including ageing, skin color, diabetes mellitus, obesity, adiposity and sarcopenia [97,98,99,100,101,102].

### 4.2. Vitamin D, Insulin Resistance and Musculoskeletal Health

Vitamin D deficiency was found to be associated with insulin resistance in a wealth of large epidemiological and cross-sectional studies [103], the strength of this association increasing with increasing BMI, being highest in subjects with obesity [104]. Several mechanisms have been called into question to explain the association between vitamin D and glucose metabolism. Via modulation of intracellular Ca^2+^ concentration, vitamin D is involved in the regulation of insulin secretion by pancreatic β cells and of membrane translocation of glucose transporters in insulin-sensitive tissues, including skeletal muscle and adipose tissue [105]. Vitamin D also plays a role both in the innate and adaptive immune system [106,107], and may exert antioxidant and anti-inflammatory effects that could counteract the inflammation driven by insulin resistance. Vitamin D modulates the gene expression of various components of the adipocyte secretome, including the adipokines such as leptin, but preclinical and clinical studies have yielded heterogeneous results on the association between vitamin D and leptin [108]. Vitamin D is also known to play a role in adipose tissue adipogenesis, lipogenesis, lipolysis and inflammation. Of note, vitamin D deficiency may affect the adipose tissue capacity of storing fatty acids, which in turn might be re-directed to other tissues, including skeletal muscle, where ectopic fat deposition may trigger inflammation and impair insulin sensitivity [109]. Consistent with preclinical evidence supporting its role in glucose metabolism and body composition, vitamin D supplementation was reported to improve insulin resistance and to reduce in total trunk fat mass in patients with obesity [110], to improve glycemic control in subjects with prediabetes [111], and to be inversely associated with insulin resistance [104], although not all studies point towards a beneficial effect of vitamin D on insulin sensitivity [112].

**Figure 2:**
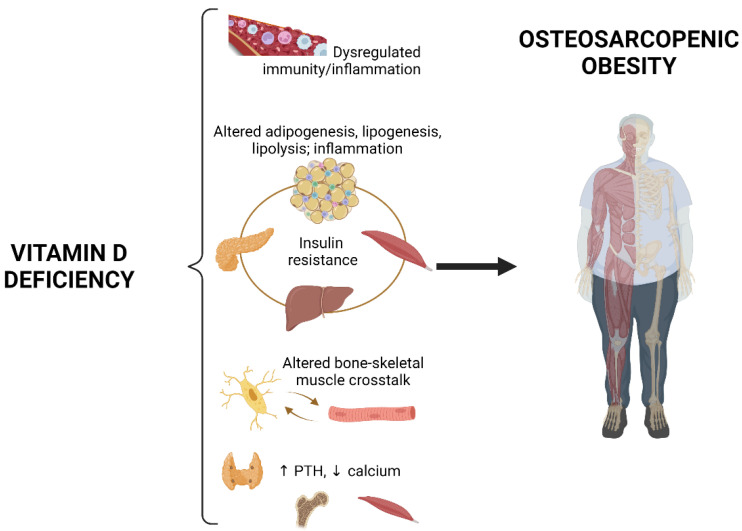
Potential role of vitamin D deficiency in the development of osteosarcopenic obesity. Vitamin D deficiency may reduce immune, antioxidant and anti-inflammatory capacity, is associated with multi-organ insulin resistance and altered adipogenesis, lipogenesis, lipolysis and inflammation in adipose tissue, might impair bone–skeletal muscle crosstalk and, by altering calcium–phosphorus metabolism, may impair muscle function and repair, chondrocyte maturation, bone mineralization and increase bone resorption. All these mechanisms could contribute (rightward arrow) to the development of osteosarcopenic obesity. PTH, parathyroid hormone. Upward arrow, increase; downward arrow, decrease. Created with BioRender.com, accessed on 31 March 2022.

Vitamin D may also be involved in the crosstalk between skeletal muscle and bone, by stimulating the production of bone- and skeletal muscle-derived factors such as osteocalcin, sclerostin, vascular endothelial growth factor (VEGF), IGF-1 and myostatin, that may act as endocrine signals between the two tissues [113]. Undoubtedly, maintaining vitamin D levels within the normal range is key to musculoskeletal health. Both direct and indirect mechanisms have been postulated, with the crucial role of vitamin D in the regulation in the calcium–phosphorus metabolism being prominent. In fact, low calcium and phosphate levels, and elevated PTH secondary to hypovitaminosis D may impair muscle function and repair, leading to proximal muscle weakness; low phosphate levels impair chondrocyte maturation and, if calcium is also low, may reduce mineralization of osteoid; high PTH levels increase bone resorption, increasing cortical bone porosity and fracture risk [114]. Direct effects of vitamin D on skeletal muscle repair and mitochondrial function have also been reported [115,116,117], as well as a protective action against IL-6-induced inflammation [118]. Pre-clinical evidence indicates that vitamin D is involved in the regulation of skeletal muscle cell proliferation, differentiation, protein synthesis and bioenergetics by modulating the expression of myogenic regulatory factors [119]. Downregulation of the VDR results in skeletal muscle atrophy and functional decline [120], while VDR overexpression has been associated with skeletal muscle hypertrophy [121]. Consistent with this evidence, individuals with severe vitamin D deficiency either due to congenital mutations or to end-stage kidney disease develop severe muscle weakness that rapidly improves after treatment with 1,25(OH)_2_D [122]. In older people with type 2 diabetes, low vitamin D intake was associated with skeletal muscle loss over approximately 14 months [123]. A large population study in older Koreans showed that vitamin D levels were significantly lower in those with low skeletal muscle mass, with or without obesity, as compared with non-sarcopenic subjects [124]. The authors found a strong correlation with vitamin D levels and the risk of sarcopenia. Similar results were reported in a subsequent cross-sectional study conducted within the framework of the Ansan Geriatric (AGE) study including 216 men and 268 women aged 65 years and older, and in another cross-sectional analysis of the Korean Sarcopenic Obesity Study (KSOS) including healthy men and women. Both studies reported lower vitamin D levels in patients with sarcopenic obesity compared to the other three groups, especially in males [125,126]. A further analysis of the Korea National Health and Nutrition Examination Survey (KNHANES), restricted to 4452 participants aged 60 years and older, including 1929 men and 2523 women, showed lower vitamin D levels in the group with sarcopenic obesity compared to the other three groups (sarcopenia, obesity and non-sarcopenia/non-obesity) and that the prevalence of sarcopenia, obesity and sarcopenic obesity increased with decreasing the dietary intake of vitamin D and protein [127]. A clinical study including patients with osteoarthritis reported seminal evidence regarding the role of leptin in sarcopenic obesity, and its relationship with vitamin D [128]. Serum leptin plays essential roles in inflammation, bone metabolism and body composition [79,80,129,130]. The authors reported that serum leptin levels positively correlated with body mass index, waist circumference, fat mass and visceral fat, and negatively with skeletal muscle index [128]. Patients with sarcopenic obesity had significantly higher serum leptin levels compared to patients with obesity alone and normal weight and, in multivariable regression models, serum leptin was negatively correlated with vitamin D. Based on the available evidence, it appears that the relationships among adipose tissue, bone and skeletal muscle are strictly entangled, and that vitamin D might play a role in modulating, at least in part, this interorgan crosstalk (Figure 2).

### 4.3. Vitamin D in Osteosarcopenic Obesity: Is There a Therapeutic Role?

Most data on the association between vitamin D and osteosarcopenic obesity are derived from large population studies from Korea. In KNHANES IV and V, including 5908 patients (2485 men, 3423 women) aged 50 years or older, high serum vitamin D level was associated with significantly lower odds of having adverse body composition features (osteopenia/osteoporosis, obesity, sarcopenia), especially osteosarcopenic obesity, in both males and females [131]. A subsequent analysis, aimed at specifically investigating the clinical manifestations and factors associated with osteosarcopenic obesity in 3267 (1080 men, 2187 women) subjects from KNHANES, confirmed that vitamin D deficiency was associated with osteosarcopenic obesity, both in men and women [37]. In fact, the prevalence of vitamin D deficiency was highest in subjects with osteosarcopenic obesity, as compared with those with obesity, sarcopenic obesity or osteopenic obesity.

Based on the favorable, pleiotropic effects of vitamin D on tissues involved in the pathogenesis of osteosarcopenic obesity, inflammation and glucose metabolism, as well as on the association between low vitamin D levels and osteosarcopenic obesity, there is a strong rationale for supplementing subjects with osteosarcopenic obesity with vitamin D. To the best of our knowledge, no studies have specifically investigated the effects of vitamin D supplementation in osteosarcopenic obesity. The best available evidence to date comes from systematic reviews and metanalyses that investigated the effect of vitamin D supplementation, at different doses and in subjects with different characteristics, on insulin resistance, body composition and indices of muscle function. A large random-effects meta-analysis and trial sequential analysis of randomized controlled trials of vitamin D in adults showed that vitamin D had no effect on total fracture (RR 1.00 [95% CI 0.94–1.07]), hip fracture (RR 1.11 [0.97–1.26]), falls (RR 0.97 [0.93–1.02]), or BMD at any site (range −0.16% to 0.76% over 1–5 years) [122]. Another systematic review and meta-analysis of randomized placebo-controlled trials showed either no difference or even worsening of muscle function tests such as the timed up and go test (0.15 [95% CI 0.03 to 0.26] more seconds spent performing the test) or the maximum knee flexion strength [−3.3 (95% CI −6.63 to −0.03) Newton] compared with placebo [132]. The studies included in this analysis were conducted in humans of any age (except athletes), supplemented with vitamin D2 or D3 *versus* placebo, regardless of mode or duration of administration, with or without calcium co-supplementation. Mean study duration was only 6 months. More recently, another systematic review and meta-analysis of placebo-controlled trials found that vitamin D monotherapy did not improve any sarcopenia indices (handgrip strength, timed up-and-go, and appendicular lean mass) in community-dwelling adults aged 50 years or older, and was associated with a significant reduction in physical performance, as assessed by the short physical performance battery score (−0.23 [95% CI −0.40 to −0.06] versus placebo) [133]. A randomized controlled study in Lebanese subjects with overweight/obesity aged 65 years or older with baseline hypovitaminosis D (10–30 ng/mL) treated for 1 year with either low (600 IU) or high (3750 IU) doses of vitamin D3 daily, in conjunction with calcium supplementation (1000 mg/day), showed no change in appendicular lean mass index, nor in adiposity indices including visceral adipose tissue [134]. When given in conjunction with protein supplementation (10–44 g/day), vitamin D (100–1600 IU/day) exhibited statistically significant beneficial effects on muscle strength, as assessed by handgrip strength (+0.38 [95% CI 0.18 to 0.47] kg versus placebo) and the sit-to-stand time (−0.25 [95% CI −0.06 to −0.43] seconds versus placebo) [135]. The co-supplementation of vitamin D and protein tended to increase muscle mass, although this effect was marginally non-significant. Finally, favorable effects on appendicular muscle mass index in sarcopenic elderly subjects have been reported when vitamin D is administered in conjunction with whey protein and leucine [136].

## 5. Conclusions

We reviewed the available literature on osteosarcopenic obesity and the potential role of vitamin D in the pathogenesis and treatment of this condition. Evidence from large observational studies and pre-clinical experiments strongly supports a role of vitamin D deficiency in the pathogenesis of osteosarcopenic obesity (Figure 2). Vitamin D has a key role in musculoskeletal health, and data indicate an association between low vitamin D levels and the risk of osteosarcopenia in subjects with obesity. However, the common belief that vitamin D improves musculoskeletal health lacks solid clinical evidence, as trials specifically aimed at assessing the effects of vitamin D supplementation in patients with osteosarcopenic obesity are not available, and trials that investigated the role of vitamin D on muscle and bone health in other patient populations showed either no or even detrimental effects. However, strong limitations of these studies are the lack of data on baseline and post-treatment vitamin D levels as well as the huge variability of vitamin D doses and length of follow-up. In fact, enrollment of vitamin D sufficient subjects at baseline may heavily influence the effect of supplementation and the use of excessive dose of the hormone may result in undesired effects [137,138]. Evidence suggests that vitamin D supplementation is beneficial to skeletal muscle when associated to protein or amino acid supplementation. Therefore, it is possible that vitamin D improves musculoskeletal health synergistically with other nutrients [139]. In fact, several other mineral and vitamin deficiencies may contribute osteosarcopenic obesity [140]. Thus, more complex nutritional strategies may be needed to counteract the syndrome. Physical activity has also been shown to exert favorable effects on body composition in sarcopenic obesity [141] and osteopenia/osteoporosis in the context of obesity [142], and should therefore be part of every intervention aimed at improving the features of osteosarcopenic obesity.

Studies are needed to clarify the role of vitamin D deficiency and supplementation in patients with osteosarcopenic obesity and low vitamin D levels, with more precise assessment of fat distribution and muscle function, besides muscle mass. In fact, in the largest studies on osteosarcopenic obesity, sarcopenia was defined using only muscle mass, and an appropriate evaluation of muscle strength was lacking. A more precise definition of the syndrome is necessary, and large studies including individuals with osteosarcopenic obesity, representative of different sex, age and race, are needed to better define the role of vitamin D in the pathogenesis and treatment of this condition.
